# The selenoprotein P/ApoER2 axis facilitates selenium accumulation in selenoprotein P-accepting cells and confers prolonged resistance to ferroptosis

**DOI:** 10.1016/j.redox.2025.103664

**Published:** 2025-05-05

**Authors:** Atsuya Ichikawa, Takashi Toyama, Hiroki Taguchi, Satoru Shiina, Hayato Takashima, Kazuaki Takahashi, Yasumitsu Ogra, Ayako Mizuno, Kotoko Arisawa, Yoshiro Saito

**Affiliations:** a Laboratory of Molecular Biology and Metabolism, Graduate School of Pharmaceutical Sciences, Tohoku University, 6-3 Aoba, Aramaki, Aoba-ku, Sendai, Miyagi, 980-8578, Japan; b Japan Society for the Promotion of Science (JSPS) Postdoctoral Fellow, Graduate School of Pharmaceutical Sciences, Tohoku University, 6-3 Aoba, Aramaki, Aoba-ku, Sendai, Miyagi, 980-8578, Japan; c Graduate School of Horticulture, Chiba University, 1-33 Yayoi, Inage-ku, Chiba, 263-8522, Japan; d Laboratory of Toxicology and Environmental Health, Graduate School of Pharmaceutical Sciences, Chiba University, 1-8-1, Inohana, Chuo-ku, Chiba, 260-8675, Japan

**Keywords:** Selenoprotein P, Apolipoprotein E receptor 2, Glutathione peroxidase, Selenium, Ferroptosis

## Abstract

The essential trace element selenium (Se) plays a significant role in redox homeostasis, while Se is very reactive and has a potent toxicity. Understanding the molecular machinery that supports Se metabolism is important for the both physiological and pathophysiological context. Incorporated Se is translated/transformed in the liver into selenoprotein P (SeP; encoded by *Selenop*), an extracellular Se carrier protein that effectively transports Se to the cells via the binding to its receptor apolipoprotein E receptor 2 (ApoER2), which is taken up by cells. The present study shows that SeP is a source of Se that accumulates intracellularly and can be utilized for prolonged periods under Se-deficient conditions. In cultured cells (RD and SH-SY5Y), glutathione peroxidase (GPX) expression induced by Se supply via the SeP/ApoER2 pathway was maintained longer during Se deficiency than inorganic Se, which was promoted by ApoER2 overexpression. SeP-deficient mice showed a faster decline in brain Se levels when fed a Se-deficient diet. Preserved GPX expression induced by this SeP/ApoER2 axis contributed to oxidative stress and ferroptosis resistance, suggesting that this redundant Se metabolism contributes to prolonged Se utilization and cytoprotection.

## 1 Introduction

Selenium (Se) is a member of the chalcogen group in the periodic table and belongs to the same family as sulfur (S), functioning in redox reactions and detoxification metabolism. Se is chemically similar to S, and plants utilize both elements for biological activities without distinguishing between them [1]. Se has a larger outermost electron orbital than S and exhibits greater redox reactivity. As a result of its higher reactivity, Se is more toxic than S, and excessive intake can cause hair loss, neurological symptoms, and brittle nails [2]. Some bacteria and animals utilize the chemical properties of Se, which are more potent than those of S, in the form of selenoproteins, and they genetically distinguish between S and Se with high specificity [3]. Se is an essential trace elements for animals, as Se deficiency leads to spermatogenesis defects, cardiomyopathy, myopathy, and neurological disorders [[4], [5], [6]]. The optimal blood concentration of Se in humans ranges from 80 to 95 μg/L [7], making it a highly sensitive nutrient that can easily become either deficient or excessive.

Human consume inorganic and organic forms of Se—such as selenocysteine (Sec) and selenomethionine (SeMet)—primarily through vegetables and meat. Following intestinal absorption, Se is transported to the liver. SeMet, a major form of organic Se in plants, is nonspecifically incorporated into proteins in place of methionine and is not directly used for selenoprotein synthesis. Occasionally, SeMet is converted to Sec through methionine metabolic enzyme and then enters the Sec biosynthesis pathway via the action of Sec lyase (Scly) [8,9]. In contrast, Sec is more redox-active and potentially toxic than SeMet but functions as a critical antioxidant. Sec is particularly enriched in fungal-type vegetables and liver meat, although the latter may also accumulate heavy metals. Se atoms can nonspecifically replace S atoms in proteins under certain conditions, suggesting that SeMet may not adequately represent the specific roles of Sec in redox regulation and selenium metabolism. Dietary Sec is directly metabolized by Scly to generate inorganic Se [8,9]. This inorganic Se is then converted to selenophosphate (Se-PO_3_^2-^) by selenophosphate synthase 2 (SEPHS2). Subsequently, O-phosphoseryl-tRNA(Sec) selenium transferase (SepSecS) uses this selenophosphate to synthesize selenocysteinyl-tRNA (Sec-tRNA^Sec^) [10]. Additionally, we and several research groups have identified peroxiredoxin 6 (PRDX6) as a novel enzyme, which promotes Se-metabolism by holding unstable inorganic Se via Cys47 and efficiently supporting using it [[11], [12], [13]]. Sec-tRNA^Sec^ corresponds to the termination codon UGA, but selenocysteine insertion sequence (SECIS)-binding protein 2 binds to a stem-loop secondary structure SECIS in the 3′ untranslated region of mRNA and inserts selenocysteine [3]. There are 25 known human selenoproteins [14], which are thought to play essential roles in biological activities, including glutathione peroxidases (GPXs) and thioredoxin reductase (TXNRD). Although these Se catabolic and anabolic metabolisms are essential for synthesizing selenoproteins, rate control of Se metabolism and its contribution to oxidative stress is little known.

Selenoprotein P (SeP; encoded by *Selenop*) is an important secreted selenoprotein responsible for Se transport throughout the body and is synthesized mainly in the liver. SeP is an extremely unique protein that holds 10 selenocysteines in one molecule. SeP is secreted and circulates throughout the body as a major selenoprotein, accounting for 53 % of the total Se in human plasma. SeP has a histidine-rich sequence and not only binds to heparin but also strongly to nickel, like His-tag [15]. We have established a method for the purification of SeP from human plasma using heparin and nickel columns and have established anti-SeP antibodies. SeP in the blood binds to receptors, Apolipoprotein E receptor 2 (ApoER2; LRP8), which belongs to the low-density lipoprotein receptor (LDLR) family expressed in various tissues and is taken up into cells through endocytosis. SeP is transported to lysosomes, degraded to selenocysteine, and then undergoes the catabolic and anabolic metabolisms to utilize Se as a selenoprotein such as GPX. However, since selenoproteins can be synthesized by the same metabolic pathway even if inorganic Se is ingested, the rationale for the necessity of SeP-mediated Se transport and supply is not fully understood. Mice deficient in the *Selenop* gene can grow on a normal diet (CE-2; containing Se derived from fishmeal), and total Se levels in organs such as the liver and kidney are unchanged compared with WT mice [16]. However, in the Se-deficient condition, Se levels in the brain and testis are reduced and neurological symptoms and spermatogenesis defects are observed [16]. This suggests that the requirement for SeP varies in specificity from organ, especially important for organ transport across the blood barrier (e.g., brain and testis). Interestingly, hepatocyte-specific deletion of *Selenop* does not significantly affect brain Se levels, unlike whole-body SeP knockout mice which show severe reductions in brain Se [17]. It has also been suggested that cells in the brain take up SeP via auto-and paracrine manner and use it for selenoprotein synthesis, called SeP cycle [18], suggesting that SeP is involved in both Se retention and transport. However, the mechanism underlying Se-preservation remains unclear.

ApoER2 is expressed in the brain, ovary, and testis [19], acts as a receptor for low-density lipoprotein receptors and also contributes to cell signaling through ligands such as reelin and ApoE [20,21]. We have identified the domain of ApoER2, which is important for SeP uptake [22]. In particular, it has been reported that ApoER2 gene-deficient mice show phenotypes (neurological disorders and male infertility) similar to those of SeP-deficient mice [23,24], it would be important to understand not only SeP but also the ApoER2 (as the SeP/ApoER2 axis) to understand Se transport and its metabolism; however, details remains unknown. SeP is thought to be degraded by the lysosome after the endocytosis mediated by ApoER2 and release selenocysteine as the selenium source, although there is an alternative pathway that is re-secreted by recycling endosomes. A lysosomal degradation-independent pathway for Se utilization has also been suggested [22], which is very interesting but remains unresolved.

In the present study, we addressed the question of why life, which is capable of synthesizing selenoproteins even from inorganic Se, utilizes the SeP/ApoER2 axis to transport and utilize Se. The present study provides a resolution to this inquiry by demonstrating that SeP, when absorbed by the ApoER2 pathway, results in Se accumulation with safe and longer rather than inorganic Se. This process preserves the long-term induction of GPX and antioxidant effects.

## 2 Materials and methods

### 2.1 Cell culture, transfection and Se-deficient medium

Human Rhabdomyosarcoma RD cells were obtained from the JCRB cell bank (JCRB9072; Ibaraki, Japan). Human neuroblastoma SH-SY5Y was obtained from KAC (EC94030304-F0; Kyoto, Japan). Both cells were cultured in DMEM (+10 % (v/v) FBS and 1 % (v/v) Penicillin-Streptomycin Mixed Solution) in a Tissue culture dish Φ100 mm and maintained in the humidified incubator with 37 °C and 5 % CO_2_. In the present study, the cells were seeded on appropriate culture plates 24 h before the experiments. FBS used in the study was purchased from Sigma (Lot.BCCC5944; SA, USA), and contained 8.3 ppb of Se evaluated by ICP-MS [25].

Plasmid for over-expression of ApoER2 used in this study and the transfection method used were previously reported [22]. RD type of ApoER2 splicing variant (ExonΔ5,15,18)) was cloned into pcDNA3.1 and transfected by polyethyleneimine.

Se-deficient medium was prepared as we reported previously [26]. Briefly, bovine serum albumin (125 mg), 10 mg/mL of Insulin (25 μL), 5 mg/mL Transferrin (50 μL), and 100 mM α-tocopherol (1 μL) were added to DMEM (50 mL) and pasteurized by 0.22 μm syringe filter and used as an aliquot of Se-deficient medium.

### 2.2 Inductively Coupled Plasma Mass Spectrometry (ICP-MS)

Pelleted cultured cells were ashed by adding 250 μL of 70 % nitric acid (sg 1.42) (143–09741; Fujifilm WAKO, Osaka, Japan) and incubation for 50 °C, overnight. Then 750 μL of MQ was added and measured by ICP-MS (Agilent 8900 Triple Quadrupole ICP-MS, Agilent, Santa Clara, CA, USA). Organs were ashed by adding 500 μL of 70 % nitric acid per 10 mg (microwave sample preparation system, ETHOS UP, Millestone-general, Kanagawa, Japan), then 1500 μL of MQ was added and measured by ICP-MS. Data were calculated as total Se ng/cell count for cultured cells or total Se ng/mg of organ for organs.

### 2.3 Purified selenoprotein P (SeP)

Human SeP was purified from human plasma as we previously reported [15]. Briefly, human plasma was subjected to the heparin column and anion-exchange column next. Elution was subjected and concentrated by Ni-agarose and the eluted sample was desalted by gel-filtration (PD10, Cytiva, Buckinghamshire, UK). Human frozen plasma was provided from the Japanese Red Cross Tohoku Block Blood Center (Human experiment approved No. 25J0012).

### 2.4 Western blotting and antibodies

The protein concentration of the sample was quantified using the DC protein assay kit (Bio-Rad, Hercules, CA, USA). The amount of protein to be loaded on sodium dodecyl sulfate-polyacrylamide gel electrophoresis (SDS-PAGE) was adjusted and applied. Subsequently, the samples were transferred to PVDF membrane (Immobilon, Merck Millipore, Darmstadt, Germany), and immersed in primary antibodies (diluted 1/2000–4000 in CanGet Solution1, Toyobo, Osaka, Japan) for 2 h at room temperature. Then the membrane was immersed in secondary antibody (1/10000 in TTBS) for 1 h at room temperature. The membrane was washed with TTBS again, chemiluminescent by ImmunoStar LD (WAKO pure chemical, Osaka, Japan), and detected by LuminoGraph I (ATTO, Saitama, Japan). After detection, the membrane was stained with Coomassie Brilliant Blue (CBB). The information for antibodies used in this study were shown in Supplemental Table 1.

### 2.5 Immunocytochemistry

Cells were seeded on 12 wells of culture plate with sterilized cover glass After the stimulation, 4 %-Paraformaldehyde Phosphate Buffer Solution was added at 300 μL/well and incubate at room temperature for 10 min. Then the cells were washed twice with PBS and treated with 300 μL/well of 0.1 % Triton X-100 in PBS for 10 min. Washed once, 1 % BSA in PBS (w/v) was added at 300 μL/well and allowed to stand at room temperature for 30 min. The solution was removed and primary antibody (human SeP (hSeP) antibody 1 μg/mL, LAMP2, EEA1, Rab7, Rab11 antibody 1/500 in 1 % BSA in PBS) was added and allowed to react overnight at 4 °C. The primary antibody was removed and washed twice with PBS, then the secondary antibody (Goat pAb to Rat IgG (Alexa Fluor® 488) preadsorbed 1/1000, Goat pAb to Rb IgG (Alexa Fluor® 594) 1/1000, Goat pAb to Ms IgG (Alexa Fluor® 594) 1/1000 in 1 % BSA in PBS) were added and reacted for 1 h at room temperature, shielded from light. The secondary antibody was removed, washed once with PBS, and treated with Hoechst 33342 solution (1/1000 in 1 % BSA in PBS) for 10 min at room temperature, washed twice with PBS and applied to glass slides in VECTASHIELD® VibranceTM Antifade Mounting Medium (Vector Laboratories, Burlingame, CA, USA). Confocal laser scanning microscopy (ZEISS LSM900 Airyscan2, Oberkochen, Germany) was used for image acquisition. The information for antibodies used in this study were shown in Supplemental Table 1.

### 2.6 Sucrose density gradient centrifugation

The sucrose density gradient fractionation was performed with slight modifications of previous reports [22,27]. Cells were seeded in tissue culture dishes Φ100 mm at 2.5 × 10^6^ cells/10 mL. After incubation for 24 h, cells were washed twice with PBS and incubated in 10 mM Tris-HCl (pH 7.5) for 1 min. The culture medium was removed, and 1 mL of Lysis buffer (Tris-HCl (pH7.5, 10 mM), EDTA 1 mM, EGTA 1 mM, Sucrose 0.25 M) was added, and the cells were homogenized by Dounce grinder on ice. The sample was centrifuged at 1000 g, 4 °C for 10 min, and the supernatant was centrifuged at 8000 g at 4 °C for 20 min. The supernatant was equilibrated with 1 mL of 100 mM Na_2_CO_3_ for 5 min, then sonicated and transferred to thin wall polypropylene tubes, 2 mL of 80 % sucrose buffer was added and mixed. Then, 4 mL of 35 % sucrose buffer and 4 mL of 5 % sucrose buffer were added in layers and centrifuged in an ultracentrifuge at 180,000 g overnight at 4 °C (total volume of the sample will be 12 mL). The separated sample (1 mL for each fraction) was collected from the top to the bottom (#1–12), and subjected to WB.

### 2.7 Animals and Se-deficient diet

The experimental plan is approved by the Support Center for Laboratory Animal and Gene Research, Tohoku University (Approval No. 2019-018-05). Mice were housed in plastic cages, up to a maximum of six mice, in a 12-h light-dark cycle at a constant room temperature (22 ± 1 °C) and humidity (55 %). Dissection was performed after inhalation of isoflurane, and whole blood samples were taken from the heart and perfused with PBS. Blood was kept on ice for at least 20 min, centrifuged (4 °C, 3000 rpm, 15 min), and serum was collected. Serum and organs were stored at −80 °C. We used *Selenop* (−/−) mice as SeP KO mice established by Hill et al., previously [16]. Because SeP homo-deficient (*Selenop* (−/−)) male mice show a reproductive dysfunction phenotype, heterozygous (*Selenop* (±)) male mice were crossed with homo-deficient (*Selenop* −/−) females for strain maintenance. For the experiment of Fig. 3B–D and Supplemental Fig. 3A-C, 7 week-old male C57BL6/J purchased from Japan CREA (Shizuoka, Japan) was used. For the experiment of Fig. 3E–H and Supplemental Fig. 3D-F, 3A-D, 7-9 week-old littermate of *Selenop* (+/+) male mice, and same-age of *Selenop* (−/−) male mice were used.

Se-deficient diets were prepared using Torula yeast (KR yeast YN23L57408, Mitsubishi Corporation Life Sciences Limited, Tokyo, Japan) as a protein source. Preparation as solid diet was performed as we previously prepared (Oriental Yeast Co., LTD. Tokyo, Japan) [28,29]. The Se contents of the food were validated by ICP-MS and 0.001 μg/kg.

### 2.8 Cell viability

After the stimulations of the cells in 96 well plate, the medium was removed and 100 μL/well of medium with 10 % alamarBlue Cell Viability Reagent (Thermo Fisher Scientific, Waltham, MA, USA) was applied and allowed to stand at 37 °C for 1–2 h. Fluorescence (λ ex/em = 545/585 nm) was then measured on a SpectrMax iD5 (Molecular Devices, San Jose, CA, USA).

### 2.9 Statistical analysis

GraphPad Prism (ver10.2.2., San Diego, CA, USA) was used for the data analysis. Welch's *t*-test was used for the comparison of two groups. A multiple comparison test (one-way ANOVA, post hoc test Dunnett's method, or Tukey's method) was used to detect significant differences between three or more groups. The data are expressed as mean ± standard deviation (S.D.) and the difference was significant when the p-value was 5 % or less. Band intensity was quantified by ImageJ 1.53i (National Institutes of Health, Bethesda, MD). Detailed sample sizes and statistical methods are shown in each figure legend.

## 3 Results

### 3.1 Evaluation of GPX induction with selenite or SeP as Se sources in RD cells

**Fig. 1:**
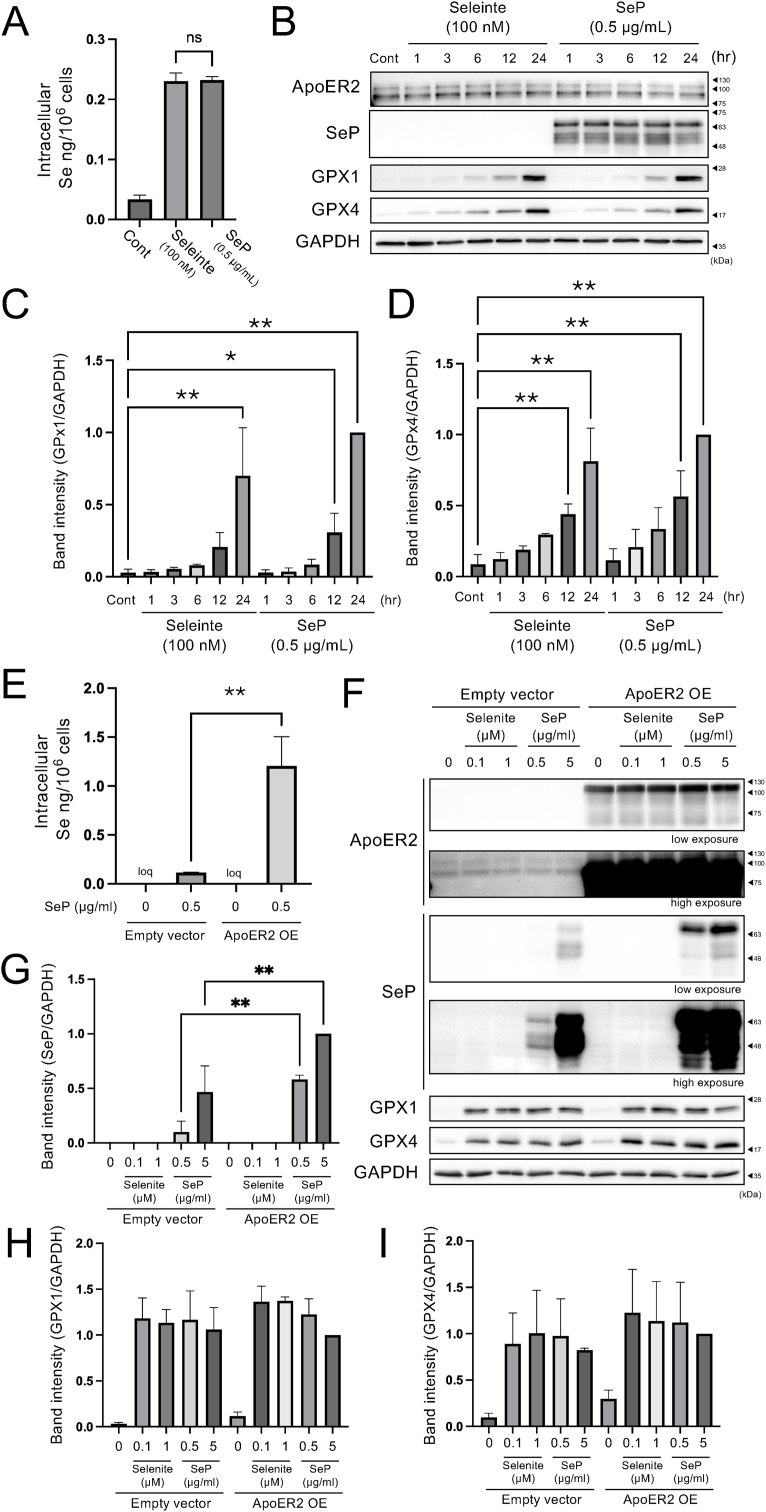
**Assessment of Se metabolism through GPX induction using selenite or SeP as Se sources in RD cells. (A)** RD cells were treated with equimolar of Se sources (selenite 100 nM or SeP 0.5 μg/mL) for 24 h. The cells were washed with PBS and collected by 0.1 % Trypsin/EDTA. After the wash of the pellets by PBS, the cells were ashed with 70 % nitric acid, and the Se content was determined by ICP-MS. Mean + S.D., n = 3, ns; not significant, Tukey's *t*-test. **(B)** RD cells were treated with selenite 100 nM and SeP 0.5 μg/mL to the indicated treatment times and WB was performed. **(C, D)** Quantitative values of the (C) GPX1 and (D) GPX4 bands of (B) were determined from triplicate repeated data independently, and corrected for GAPDH, respectively, and shown relative to the 24 h point of SeP 0.5 μg/mL as 1. Mean + S.D., n = 3, vs control, ∗P < 0.05, ∗∗P < 0.01, Dunnett's test. **(E)** ApoER2 expressing plasmid (ApoER2 OE) or empty vector was transfected to RD cells for 24 h. After that the cells were treated with selenite 100 nM and SeP 0.5 μg/mL for 24 h and the Se content was determined by ICP-MS. Mean + S.D., n = 3, ns; ∗∗P < 0.01, Tukey's *t*-test. loq indicates below limit of quantification. **(F)** ApoER2 transfected cells were treated with the indicated concentrations of selenite and SeP and WB was performed. The band of ApoER2 and SeP were too high to evaluate accurately, thus low exposure and high exposure were shown. **(G, H, I)** Quantitative values of the (G) SeP, (H) GPX1 and (I) GPX4 bands were determined from triplicate independent data, corrected for GAPDH, respectively, and shown relative to the point of ApoER2 OE, SeP 5 μg/mL as 1. The band intensity of SeP were acquired from low exposure images. Mean + S.D., n = 3, vs control, ∗P < 0.05, ∗∗P < 0.01, Dunnett's test.

GPX, as a selenoprotein that increases in a Se supply-dependent manner, is a useful marker of Se metabolism in cells. To understand the characteristics of the SeP-mediated Se transport pathway, we first compared the concentration dependence of selenite and SeP in inducing GPX expression in human rhabdomyosarcoma RD cells, a model cell line for SeP uptake via ApoER2 [22]. After 24 h of treatment of RD cells with two Se sources, selenite (100 nM) or SeP (0.5 μg/ml, approximately 10 nM of protein), the same amount of cellular Se was detected by ICP-MS analysis (Fig. 1A). Based on this result, we further checked the time dependence of GPX induction and found that both selenite and SeP induced GPX1/4 from 12 to 24 h, showing no difference between the two Se sources (Fig. 1B–D). Cellular SeP was detected after 1 h of treatment and remained constant for 24 h, indicating that SeP is rapidly taken up and stored in cells. At the same time, it is considered that there is a limit to the cellular SeP amount that can be retained. Interestingly, overexpression of ApoER2 broke this limit, and SeP uptake increased approximately 10-fold as assessed by intercellular Se and SeP protein levels (Fig. 1E–G), in contrast, selenite did not change its cellular uptake by ApoER2 OE (Supplemental Fig. 1). The induction of GPX1/4 was not altered compared to the control (Fig. 1F–H, and I). These results suggest that SeP and selenite in equal cellular Se amounts are used for the synthesis of cellular selenoproteins; however, selenoprotein synthesis is regulated separately from Se uptake. The results also indicated that SeP supercharged by the SeP/ApoER2 axis accumulates in cells without undergoing subsequent degradation. Thus, we hypothesized that if SeP is taken up by ApoER2, it might have Se storage properties in the cell and verified the subcellular localization and persistence of cellular selenoproteins under Se deficient conditions.

### 3.2 Subcellular localization of incorporated SeP in the cells

We next check the subcellular localization of SeP under control and ApoER2-overexpression condition. SeP has an ApoER2 binding motif in its C-terminal region and is incorporated into the cells by endocytosis. Previously, we verified subcellular localization in Jurkat and RD cells treated with SeP for 6 h and found accumulation in various fractions, including lysosomes. Therefore, immunostaining of endosomal and lysosomal markers (early endosome antigen1; EE1A, Ras-related protein7; RAB7, Ras-related protein11; RAB11, and lysosomal associated membrane protein 2; LAMP2) and SeP was performed to confirm their co-localization by confocal microscopy imaging.

**Fig. 2:**
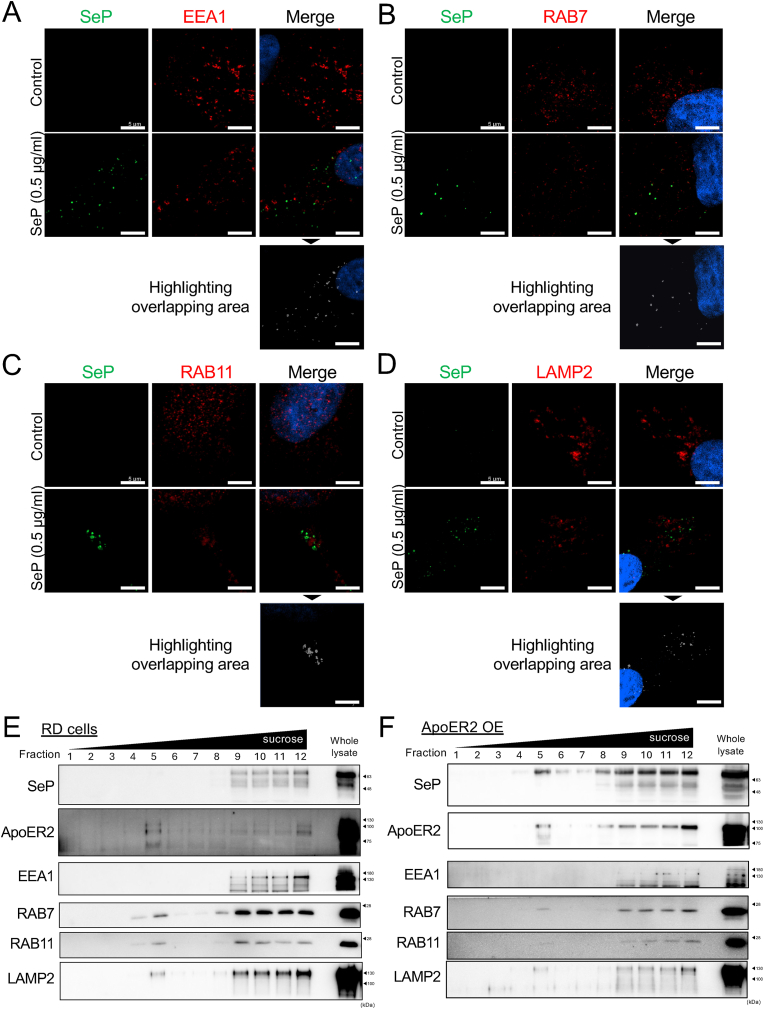
**Subcellular localization of SeP in RD cells. (A)** RD cells were seeded on the cover glass and grown for 24 h. SeP (0.5 μg/mL) was treated for 24 h and stained with EEA1 (red), SeP (green) and Hoechst (blue). **(B)** The cells were stained with RAB7 (red), SeP (green) and Hoechst (blue). **(C)** The cells were stained with RAB11 (red), SeP (green) and Hoechst (blue). **(D)** The cells were stained with LAM2 (red), SeP (green) and Hoechst (blue). The scale bar indicates 5 μm. **(E)** RD cells or ApoER2 over-expressing RD cells **(F)** were treated with SeP 0.5 μg/mL for 24 h and cell lysates were separated using density gradient centrifugation. Each fractions were subjected to WB.

After treatment of the cells with 0.5 or 5 μg/ml of SeP for 24 h, SeP was colocalized with all the endosomal and lysosomal markers (Fig. 2A–D, and Supplemental Fig. 2). An enlarged figure showing a more distinct subcellular localization by treating cells with SeP 5 μg/ml is presented in Supplementary Fig. 2. The cell fractions containing SeP were further evaluated by density gradient centrifugation and found to be more abundant in Fraction #12, which contains the largest part of lysosomes (Fig. 2E). SeP was found in early endosomes and late endosomes, which was indicated by EEA1 and Rab7 as well. Immunostaining showed co-localization of a part of SeP with Rab11, which indicates recycling endosomes. However, there was only a small accumulation in fraction #5, which concentrates on recycling endosomes [22,27]. In contrast, when ApoER2 was over-expressed, SeP was detected in endosomal and lysosomal fractions, including the recycling endosomes (Fig. 2F). Two bands of SeP, at 65 kDa and 50 kDa, indicate a glycosylated-intact form and a truncated form by the kallikrein cleavage site. Particularly, an intact SeP of 65 kDa was predominantly detected in the recycling endosome, suggesting that there may be intracellular segregation machinery for intact and truncated SeP, although the details of this mechanism are unknown. Taken together, these results suggest that SeP distributes to endosomal and lysosomal fractions, including the recycling endosomes, which were enhanced by the ApoER2 overexpression.

**Fig. 3:**
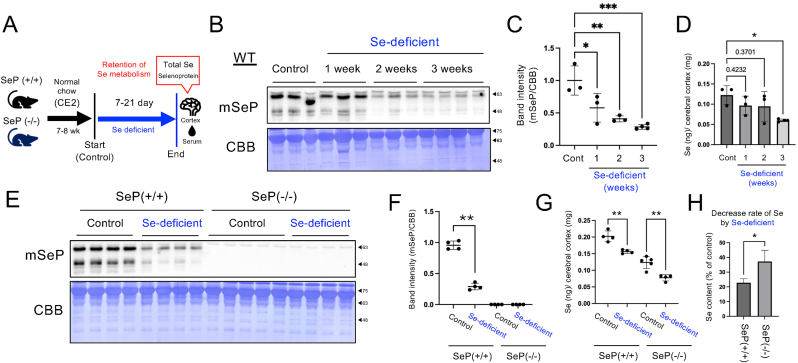
**Effect of Se-deficiency on Se-metabolism in SeP knockout mice. (A)** Experimental design of animal experiment with Se-deficiency. **(B)** WT mice grown on normal diet (CE2) were fed a Se-deficient diet for 1–3 weeks and plasma SeP expression levels were checked by WB. **(C)** WB of serum; quantitative values of SeP were determined and corrected for CBB, respectively. Mean ± S.D., n = 3–4, vs CE2, ∗P < 0.05, ∗∗P < 0.01, ∗∗∗P < 0.001, Dunnett's test. **(D)** The cerebrum cortex was ashed with 70 % nitric acid, and the Se content was determined by ICP-MS. Mean ± S.D., n = 3–4, ∗P < 0.05, Dunnett's test. **(E)** WT and SeP KO mice grown on normal diet (CE2) were fed a Se-deficient diet for 2 weeks and collected the blood and serum was obtained. WB for mouse selenoprotein P (mSeP) were performed. **(F)** Band quantification values of mSeP were determined and corrected for Coomassie Brilliant Blue (CBB) staining, respectively. Mean ± S.D., n = 4–5, ∗∗P < 0.01, Welch's *t*-test. **(G)** The cerebrum cortex or WT and SeP KO mice were ashed with 70 % nitric acid, and the Se content was determined by ICP-MS. Mean ± S.D., n = 4–5, ∗P < 0.05, ∗∗P < 0.01, Tukey'test. **(H)** Data from D quantified the rate of decrease in Se content due to 2 weeks of Se deficiency. Mean ± S.D., n = 4–5, ∗P < 0.05, Welch's *t*-test.

### 3.3 Effect of Se deficiency on the Se contents and GPX levels in the brain of SeP deficient mice

To address the hypothesis that SeP is involved in Se retention as well as transport, SeP KO mice were fed a Se-deficient diet, and Se persistence of SeP was tested based on organ Se levels and GPX expression levels (Fig. 3A). The total Se level in the normal chow diet (CE-2) was 0.05 mg/kg, and the Se-deficient diet was 0.001 μg/kg as quantified by ICP-MS.

First, WT C57BL6/J mice were fed a Se-deficient diet for 1–3 weeks. It has been reported that SeP is preferentially incorporated in the brain when administered peripherally [30]. Therefore, the cerebrum, where SeP-dependent Se transport is active, was selected as the organ of interest for analysis. Results showed that after switching to a Se-deficient diet, SeP in serum decreased within 1 week (Fig. 3B and C), and cerebral Se levels decreased within 2–3 weeks (Fig. 3D). Although cerebral Gpx4 did not show significant changes with Se deficiency, Gpx1 showed a decreasing trend at week 3 (Supplemental Fig. 3A–C). Therefore, a comparison was made with SeP KO (*Selenop*(−/−)) and its littermate *Selenop*(+/+) mice after 2 weeks of Se-deficient food intake when the brain became Se-deficient.

SeP KO mice were fed a Se-deficient diet for 2 weeks and serum SeP levels were evaluated by Western blotting. As a result, serum SeP was completely abolished under basal conditions in SeP KO mice, and no effect of the Se-deficient diet (Fig. 3E and F). Gpx1 expression in the cerebrum of SeP KO mice was reduced under control conditions and was not altered by Se deficiency in either mouse (Supplemental Fig. 3D and E). The reduction of Gpx4 by a Se-deficient diet was also not clear in Selenop (+/+) and SeP KO mice (Supplemental Fig. 3D and F). Interestingly, a reduction of total Se in the brain was also found on the Se-deficient diet (Fig. 3G), but the ratio of this reduction was significantly greater in the SeP KO than in the *Selenop* (+/+) mice (Fig. 3H). The testis is another organ that is highly dependent on Se transport by SeP, and SeP KO mice show failure of spermatogenesis. In the testis, total Se levels and Gpx expression were not altered by a 2-week Se-deficient diet (Supplementary Fig. 4A–D). Total Se levels were very low at the basal level in SeP KO mice (Supplemental Fig. 4A), and there was no change in Gpx levels within a 2-week Se-deficient diet. These results suggest that the testis may rely dominantly on SeP for Se transport.

Taken together, SeP may be partly involved in Se retention in the brain under Se deficient conditions, in addition to Se transport, although the mixed effects of SeP on Se transport and retention were difficult to evaluate separately *in vivo*. Therefore, a study of Se metabolic retention in cultured cells was further performed.

### 3.4 Preservation of GPX by SeP/ApoER2 axis under Se-deficient conditions

**Fig. 4:**
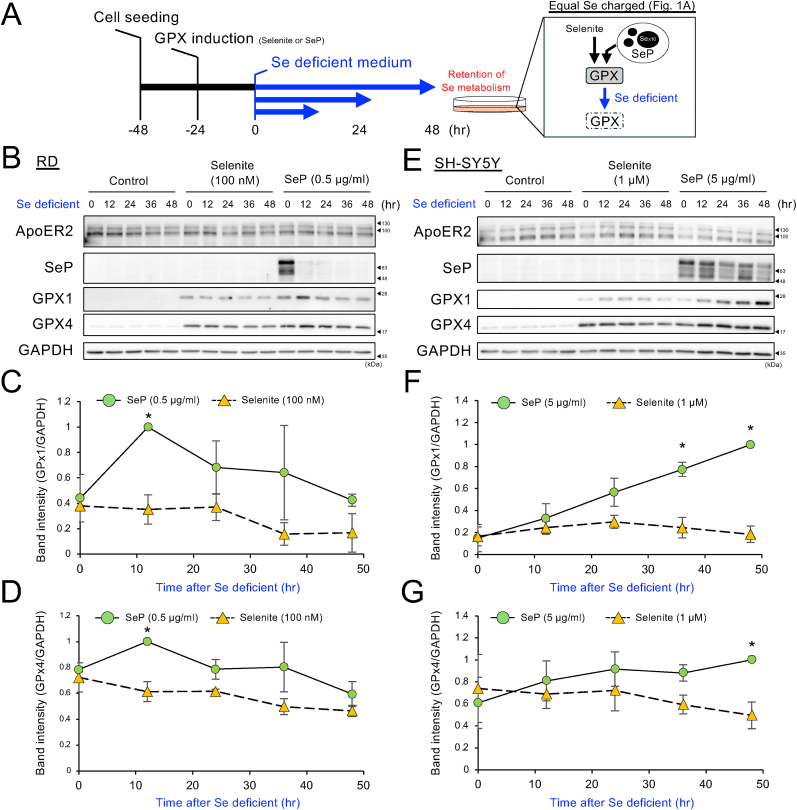
**Expression of GPX as an assessment of Se metabolism in Se-deficient conditions. (A)** Experimental scheme for Se-deficient experiments. Assess the reduction of GPX by placing GPX-induced cells in a Se-deficient state. **(B)** RD cells were treated with selenite 100 nM and SeP 0.5 μg/mL for 24 h. The medium was replaced with Se-depleted medium and further incubated for indicated time period. After that the cells were harvested and WB performed. **(C, D)** Quantitative values of the (C) GPX1 and (D) GPX4 bands were determined from three independent data sets, corrected for GAPDH, respectively, and shown relative to the SeP 12 h time point as 1. Mean ± S.D., n = 3, SeP vs selenite in each time point, ∗P < 0.05, Welch's *t*-test followed by Bonferroni correction. **(E)** SH-SY5Y cells were treated with selenite 1 μM and SeP 5 μg/mL for 24 h. The medium was replaced with Se-depleted medium and further incubated for indicated time period. After that the cells were harvested and WB performed. **(F, G)** Quantitative values of the (F) GPX1 and (G) GPX4 bands were determined from three independent data sets, corrected for GAPDH, respectively, and shown relative to the SeP 48 h time point as 1. Mean ± S.D., n = 3, SeP vs selenite in each time point, ∗P < 0.05, Welch's *t*-test followed by Bonferroni correction.

To assess the prolonged expression of GPXs by SeP/ApoER2 axis, RD cells were treated with equimolar of Se (100 nM selenite or 0.5 μg/mL SeP) for 24 h and charged equal Se in the cells (Fig. 1A). After that, the incubation medium was changed to a Se-deficient medium, and a time-dependent decrease in GPX expression was evaluated as the persistence of Se-metabolism (Fig. 4A).

First, selenite-treated cells were incubated under a Se-deficient medium and a time course of decrease in GPX was examined. As a result, a GPX reduction of about 50 % was observed within 48 h and reached almost basal within 96 h in RD cells (Supplemental Fig. 5). Next, the persistence of GPX levels was compared between selenite and SeP within 48 h. The results indicated that when GPX1/4 were induced by SeP, both expressions were significantly increased after Se-deficiency within 12 h and then decreased (Fig. 4B–D). SeP charged in the cells was significantly reduced during the first few hours after Se deficiency (Supplemental Fig. 7), which may have been compensated for increased GPX expression. This reduction in GPX was not suppressed by adding selenite to the Se-deficient medium, suggesting that the system does not sense Se deficiency and actively degrades it. A mechanism dependent on the outer SeP concentration may be assumed. In addition, since not much SeP was re-released from SeP-charged cells into the Se-depleted medium (Supplemental Figs. 7 and 8), the main mechanism likely is that SeP undergoes degradation inside the cells. The SeP, which declined rapidly within a few hours, may be stored in the form of partially degraded peptides rather than as intact protein. A similar study was carried out in human neuroblastoma cells, SH-SY5Y. However, under the same concentration conditions as RD, no long-term Se utilization effect was observed. Therefore, a comparative study with 1 μM selenite and 5 μg/ml SeP (100 nM), which are Se equivalents, showed more pronounced differences, with basal GPX expression not being reduced by Se deprivation, but GPX induced with SeP continuing to increase in a time-dependent manner under Se deprivation (Fig. 4E–G). In SH-SY5Y cells, the decrease in SeP due to Se deficiency was mild compared with RD. Furthermore, ApoER2-overexpressing RD cells showed a marked increase in SeP accumulation, and GPX continued to increase up to 48 h despite Se deficiency in RD cells (Supplemental Fig. 6).

Collectively, these data show for the first time that SeP is a Se source with a higher "persistence of bioavailable Se" than selenite, and ApoER2 was implicated in this redundant Se metabolism.

### 3.5 Acquisition of prolonged resistance to oxidative stresses in Se-deficient conditions by SeP

**Fig. 5:**
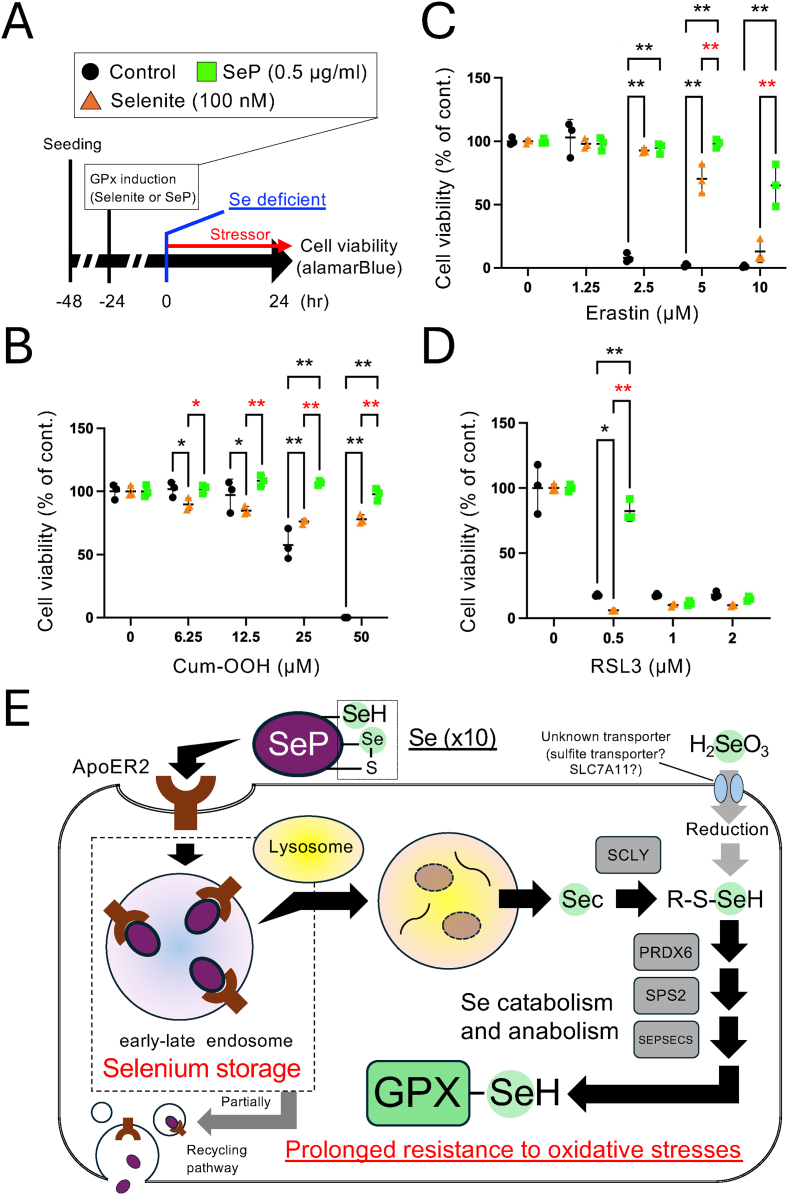
**Contribution of SeP in the defense against ferroptosis in Se deficient states. (A)** Experimental scheme to evaluate ferroptosis resistance in Se-deficient conditions. **(B, C, D)** RD cells were seeded in 96 well plates and treated with selenite 100 nM and SeP 0.5 μg/mL for 24 h. After that, the cells were treated with Se-deficient medium containing stressors, (B) Cumenehydroperoxide (Cum-OOH), (C) Erastin and (D) RSL3 for 24 h. The cell viability was determined using alamarBlue assay. Mean ± S.D., n = 3, ∗P < 0.05, ∗∗P < 0.01, Tukey's multiple comparisons test. **(E)** Summary of this study. SeP taken up via ApoER2 are degraded and utilized via storage vesicles and are involved in the retention of GPX induction for long periods even under Se-deficient conditions, and repress oxidative stresses e.g., ferroptosis.

GPXs are involved in the defense against oxidative stress. In particular, GPX4 is involved in reducing phospholipid hydroperoxides and has recently been known as a ferroptosis regulator. In the present study, since SeP was found for the first time to be involved in the prolonged expression of GPX4, we investigated its sensitivity to ferroptosis and lipid hydroperoxides under Se-deficient conditions (Fig. 5A).

According to the above, RD cells were treated with equal amounts of selenite and SeP for 24 h to charge the cells with the same amount of Se. Then, the medium was changed to a Se-deficient medium with cumene hydroperoxide (Cum-OOH) or ferroptosis inducers (Erastin and RSL3). These stressors induced cell death, and it was partly protected by selenite, but SeP was more protective (Fig. 5B–D). This is consistent with the expression of GPX4, suggesting that SeP exerted this protective effect due to its long-term GPX4 retention (Fig. 5C and D). Given that intracellular Se and GPX expression levels were similar at baseline in selenite and SeP (Fig. 1), this difference may have been caused by the ability of SeP to maintain long-term GPX expression.

These results indicate that SeP is involved in the acquisition of resistance to oxidative stress by promoting Se-metabolism during Se deficiency through the formation of Se-storage in the cells (Fig. 5E).

## 4 Discussion

This study suggests that 1. SeP was not only a source of Se but also accumulated intracellularly upon ApoER2 expression. 2. Stored SeP was degraded in lysosomes during selenium deficiency and contributed to the continuous expression of GPX, and 3. This Se storage system was involved in antioxidant system under Se-deficient conditions. In conclusion, the present study suggests that SeP is not a simple Se-carrier protein and plays an essential role in maintaining unstable Se-metabolism.

The transporter of selenite has not been identified, but it has been suggested that SLC7A11 is partially responsible for the uptake of selenite reacting with extracellular cysteine [13]. Selenite is reduced by glutathione in cells and metabolized to selenodiglutathione (GS-Se-SG) and hydrogen selenide (H_2_Se), and nucleophilic attack on proteins is thought to be the mechanism of toxicity [31,32]. In addition, oxidative DNA damage and generation of reactive oxygen species (ROS) by Se have been reported as mechanisms of toxicity [33]. Thus, reactions with protein thiols are thought to be implicated in exerting Se toxicity, but selenocysteine residues in SeP has been reported to form seleneylsulfide bonds (R-Se-S-R) with thiol in vicinal cysteine [34], which is expected to inhibit chemical reactivity. It is, therefore, possible that the cytotoxicity of SeP may be lower than that of inorganic Se, and we thought this might be another reason why inorganic Se and SeP are distinguished as Se sources. However, as 100 nM of selenite was not toxic under the conditions for evaluating Se metabolism, the concentrations of SeP and selenite were increased to toxic levels. The results showed that in the toxic range, selenite was more likely to accumulate in cells and induce cell death than SeP at equimolar as Se (Supplemental Fig. 9A and B). Notably, the amount of Se accumulated at this toxic condition was approximately 0.5 ng/10^6^ cells, whereas when SeP was taken up by over-expression of ApoER2, the amount of Se was more than twice as high (1.2 ng/10^6^ cells) (Fig. 1E), but no toxicity was observed. These results suggest that, unlike inorganic Se, SeP can be a safe saving of Se in the cells. Selenite is thought to be distributed in the cytoplasm after uptake, whereas in the case of selenoprotein P, selenium is thought to be localized to endosomes and lysosomes. Therefore, this difference in intracellular distribution of selenite and SeP may also account for the difference in toxicity. As tissues, total selenium concentrations are higher in kidney and liver, but the ratio of inorganic to organic selenium and the spatial understanding of local intracellular selenium levels in cells with high levels of receptors such as ApoER2 may explain such toxicity differences and should be explored future.

In the liver, a major organ for SeP production, ApoER2 expression is low, and the balance is biased toward secretion. In cells that form SeP cycles, both SeP expression and receptor expression could be high, for example, astrocytes in the brain. At Fig. 4, relatively higher concentrations were required to observe selenium storage by SeP in SH-SY5Y cells compared to RD cells. As we previously reported, SH-SY5Y cells seem to have a mechanism to extract selenium without degrading SeP, which may have required a relatively high concentration to evaluates Se preservation, thus it is possible that the storage of SeP may be a mechanism specialized for degradation [22]. Future studies may reveal the mechanism of the SeP cycle in the brain by focusing on the receptors and the characteristics of Se utilization without breakdown of SeP.

Overexpression and over-accumulation of SeP into the cells is not only a good thing. Excess SeP expression in glioblastoma also leads to autocrine retention of Se metabolism, which may contribute to treatment resistance [35]. We have shown that excess SeP is involved in pulmonary hypertension and diabetes as well [36,37]. Perhaps this excessive accumulation of SeP storage vesicles could change redox status and cellular functions, but in this study, we did not observe any cell death at this time scale. Longer incubation periods and assessment of cell function and viability could allow the study of the various adverse effects associated with SeP overaccumulation. The use of RD cells and undifferentiated nerves in this study did not allow functional assessment of the cells, which is one of the limitations of this study.

The relationship between ApoER2 expression and diseases described above is little understood. Thus, future studies on cellular dysfunction caused by hyper-accumulation of SeP by the ApoER2 system are needed. Known regulators of ApoER2 expression levels include NF-E2-related factor 2 (NRF2) [38], sorting nexin-17 (SNX17) [39], inducible degrader of the LDL receptor (IDOL) [40], and proprotein convertase subtilisin kexin 9 (PCSK9) [41]. SNX17 binds to the intracellular domain of ApoER2 and regulates ApoER2 recycling via transport from early endosomes to recycling endosomes, positively regulating its expression at the cell surface. As IDOL is known to be expressed downstream of LXRs, likely, such factors are also involved in Se metabolism through the regulation of ApoER2 expression [42]. Since IDOL is known to be expressed downstream of LXR, treatment of RD cells with the LXR agonist GW3965 showed partial inhibition of SeP accumulation with a decrease in ApoER2, although not enough to reduce GPX induction by SeP in the cells (Supplemental Fig. 10). Previously, we have found that NRF2 activator sulforaphane inhibits SeP expression in HepG2 cells and mouse plasma. However, this has been explained by the inhibition of Se transport activity via the promotion of SeP degradation and covalent binding of selenocysteine residues of SeP [43,44]. Behind this action, NRF2 may have a hidden function to promote SeP uptake via increased ApoER2 expression.

As mentioned in the introduction, TXNRD is one of the selenoproteins important for life support. We evaluated the same reduction in TXNRD in selenium-deficient media as in GPX, but at 48 h there was little reduction and no difference between selenite and SeP (Supplemental Fig. 11). This may be due to the high SECIS hierarchy order of TXNRD, which may have been relatively tolerant to selenium deficiency [45], and the possibility that TXNRD mRNA is resistant to nonsense-mediated RNA decay (NMD) and truncated TXNRDs are synthesized without selenium [46]. For these reasons, TXNRD seems not a good indicator of selenium metabolism.

The subcellular localization of SeP was examined using fluorescent immunostaining and sucrose density gradient centrifugation and revealed that the intracellularly accumulated SeP was widely distributed along the endosomal-lysosomal pathway. This was also observed at multiple time points: SeP uptake was maximal at 1 h, and conversely, when SeP was removed from the medium, SeP was degraded within a few hours. Thus, it appears that cells take up SeP and retain it in the endosomal-lysosomal pathway while degrading it rapidly, and it may be stored in a peptide state containing selenocysteine rather than SeP, for example. Analysis of these undigested products is difficult and is the limitation of this study.

Taken together, the present study suggested SeP/ApoER2 axis has a unique Se storage function in the cells. The findings may also lead to an understanding of the acquisition of robustness for Se-metabolism in Se-deficient regions and patients dependent on intravenous complete nutrition. It may also be important to understand the possibility of high ApoER2 expression and unintended SeP overaccumulation as a drug action, as PCSK9 inhibitors are already used as clinical agents for hyperlipidemia [47]; the relationship between SeP excess and disease may also have implications for drug development. SeP receptors are known to be ApoER2 as well as LRP1 and megalin. The importance of considering Se metabolism from the receptor side is little understood, and this perspective may reveal interesting redox-regulatory actions.

## 5 Conclusion

This study demonstrated that Selenoprotein P (SeP), taken up via ApoER2, acts not only as a Se transporter but also as an intracellular reservoir under Se-deficient conditions. SeP sustains glutathione peroxidase (GPX) expression, enhancing cellular resistance to oxidative stress and ferroptosis. In SeP-deficient mice, Se depletion in the brain progressed rapidly under Se-deficient diets, highlighting SeP essential role in maintaining Se homeostasis and antioxidant defense. These findings suggest that the SeP/ApoER2 axis is crucial for cellular protection during Se deficiency.

## CRediT authorship contribution statement

**Atsuya Ichikawa:** Data curation, Investigation, Visualization. **Takashi Toyama:** Conceptualization, Funding acquisition, Investigation, Project administration, Supervision, Visualization, Writing – original draft, Writing – review & editing. **Hiroki Taguchi:** Investigation, Writing – review & editing. **Satoru Shiina:** Investigation. **Hayato Takashima:** Investigation, Resources. **Kazuaki Takahashi:** Methodology, Resources. **Yasumitsu Ogra:** Methodology, Resources. **Ayako Mizuno:** Investigation, Methodology. **Kotoko Arisawa:** Resources. **Yoshiro Saito:** Funding acquisition, Resources, Supervision, Writing – review & editing.

## Availability of data and materials

The authors declare that all data are available within the article under reasonable requirements. The materials e.g., SeP antibodies are also available on request.

## Ethics approval and consent to participate

All experimental procedures were conducted in accordance with the Tohoku University. The animal study was carried out in accordance with the rules and guidelines for the proper implementation of animal experiments at Tohoku University and the experimental plan is approved by the Support Center for Laboratory Animal and Gene Researches, Tohoku University (Approval No. 2019-018-05).

## Funding

This study was supported in part by part by JSPS KAKENHI (grant number 21K19321 for YS and 23H03546 for TT)

## Declaration of competing interest

The authors declare the following financial interests/personal relationships which may be considered as potential competing interests: Takashi Tyama reports financial support was provided by 10.13039/501100001691Japan Society for the Promotion of Science. Yoshiro Saito reports financial support was provided by japan society for the promotion of science. If there are other authors, they declare that they have no known competing financial interests or personal relationships that could have appeared to influence the work reported in this paper.
