# Supplementary material for: The selenoprotein P/ApoER2 axis facilitates selenium accumulation in selenoprotein P-accepting cells and confers prolonged resistance to ferroptosis
Source: Redox Biol. 2025 May 5;83:103664. doi: 10.1016/j.redox.2025.103664 (PMC12138407; doi:10.1016/j.redox.2025.103664)
Supplement: Multimedia component 1 [file mmc1.pdf]

Redox Biology

# The selenoprotein P/ApoER2 axis facilitates selenium accumulation in selenoprotein P-accepting cells and confers prolonged resistance to ferroptosis

Supplemental Table 1.

| <Primarly antibodies> | Catalog No |
|---|---|
| Rat monoclonal SeP (human specific) BD1 antibody | Original |
| Rabbit monoclonal Anti-ApoER2 antibody | ab108208, abcam |
| Rabbit monoclonal Anti-GPX1 antibody | ab108427, abcam |
| Rabbit monoclonal Anti-GPX4 antibody | ab125066, abcam |
| Mouse monoclonal Anti-LAMP2 antibody | sc-18822, Santa Cruz |
| Mouse monoclonal Anti-EEA1 antibody | M176-3, MBL |
| Mouse monoclonal Anti-Rab7 (B-3) antibody | sc-376362, Santa Cruz |
| Rabbit monoclonal Anti-Rab11 antibody | 5589S, Cell Signaling |
| Rabbit monoclonal Anti-Cathepsin B antibody | 31718, Cell Signaling |
| Mouse monoclonal Anti-GAPDH Peroxidase conjugated | 015-25473, Wako |
| Rabbit polyclonal Anti-mouse SeP (mSeP) antibody | Original |
| Rabbit monoclonal TXNRD1 antibody | 15140, Cell Signaling |

| <Secondary antibodies> | Catalog No |
|---|---|
| Goat polyclonal Anti-Rabbit Immunoglobulins/HRP | P0448, Dako |
| Goat polyclonal Anti-Rat Immunoglobulins/HRP | P0450, Dako |
| Goat polyclonal Anti-Mouse Immunoglobulins/HRP | P0447, Dako |
| Goat Anti-Rat IgG H&L (Alexa Fluor® 488) preadsorbed | ab150165, abcam |
| Goat Anti-Mouse IgG H&L (Alexa Fluor® 594) | ab150116, abcam |
| Goat Anti-Rabbit IgG H&L (Alexa Fluor® 594) | ab150080, abcam |

**Supplemental Figures**

# Supplemental Figure 1

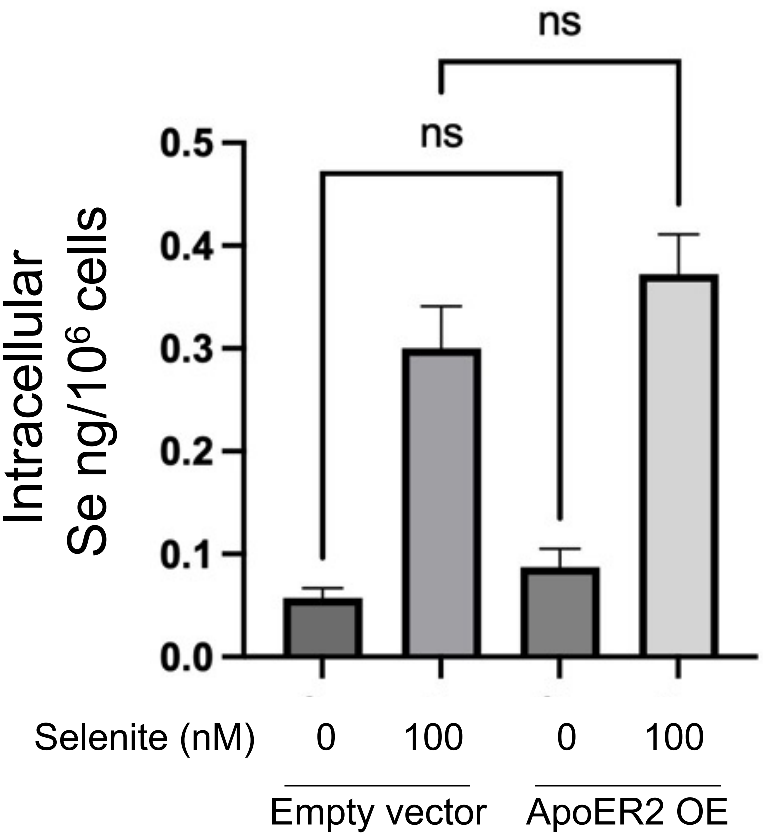


**Supplemental Figure 1. Intracellular Se level of ApoER2-OE cells treated with selenite.** RD cells were transfected with empty vector or ApoER2 expressing plasmid (ApoER2 OE), then the cells were treated with selenite (100 nM) for 24 hr. Then the cells were washed and subjected to ICP-MS. Mean ± S.D., Tukey'test. n=3. n.s. indicates not significant.

# Supplemental Figure 2

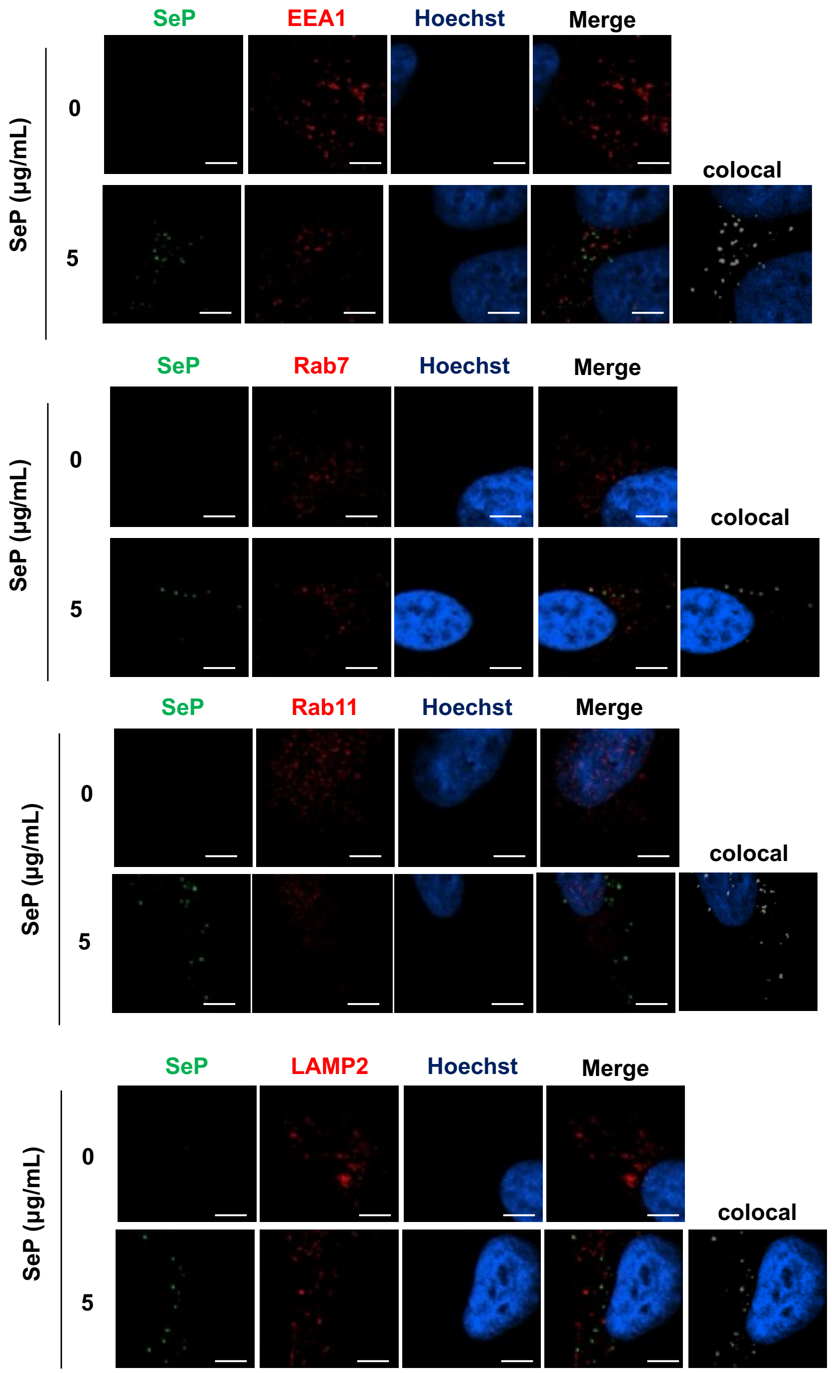


**Supplemental Figure 2. Subcellular localization of SeP in RD cells.** RD cells were seeded on the cover glass and grown for 24 hr. SeP (0.5 or 5 μg/mL) was treated for 24 hr and stained with each antibody (red), SeP (green), and Hoechst (blue). The scale bar indicates 10 μm. Marged areas were emphasized by white. Images of control are the same as Figure 2, and the images were taken at the same timing and with the same fluorescence intensity.

# Supplemental Figure 3

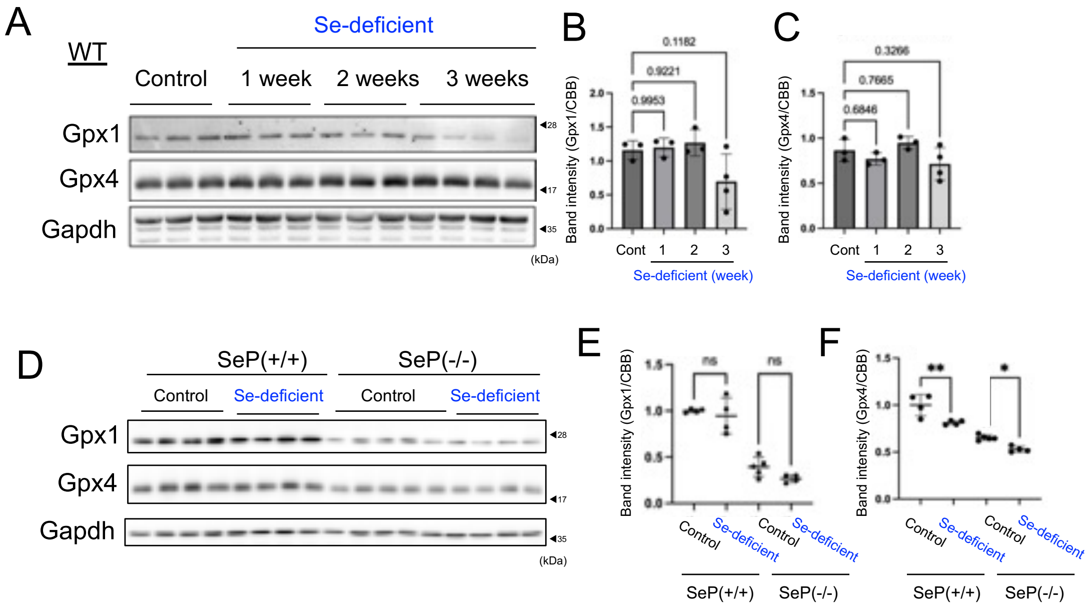


**Supplemental Figure 3. Effect of Se-deficiency on brain Gpx in mice.** WT Mice were fed a Se-deficient diet. **(A)** WB of cerebrum cortex; Quantitative values of the **(B)** GPX1 and **(C)** GPX4 bands were corrected for each GAPDH. Mean ± S.D., Dunnett's test. WT and SeP KO mice grown on normal diet (CE2) were fed a Se-deficient diet for 2 weeks. **(D)** WB of cerebrum cortex of WT or SeP KO mice shown in. **(E, F)** Quantitative values of the (E) GPX1 and (F) GPX4 bands were corrected for each GAPDH. Mean ± S.D., n=4-5, *P<0.05, **P<0.01, Tukey'test.

# Supplemental Figure 4

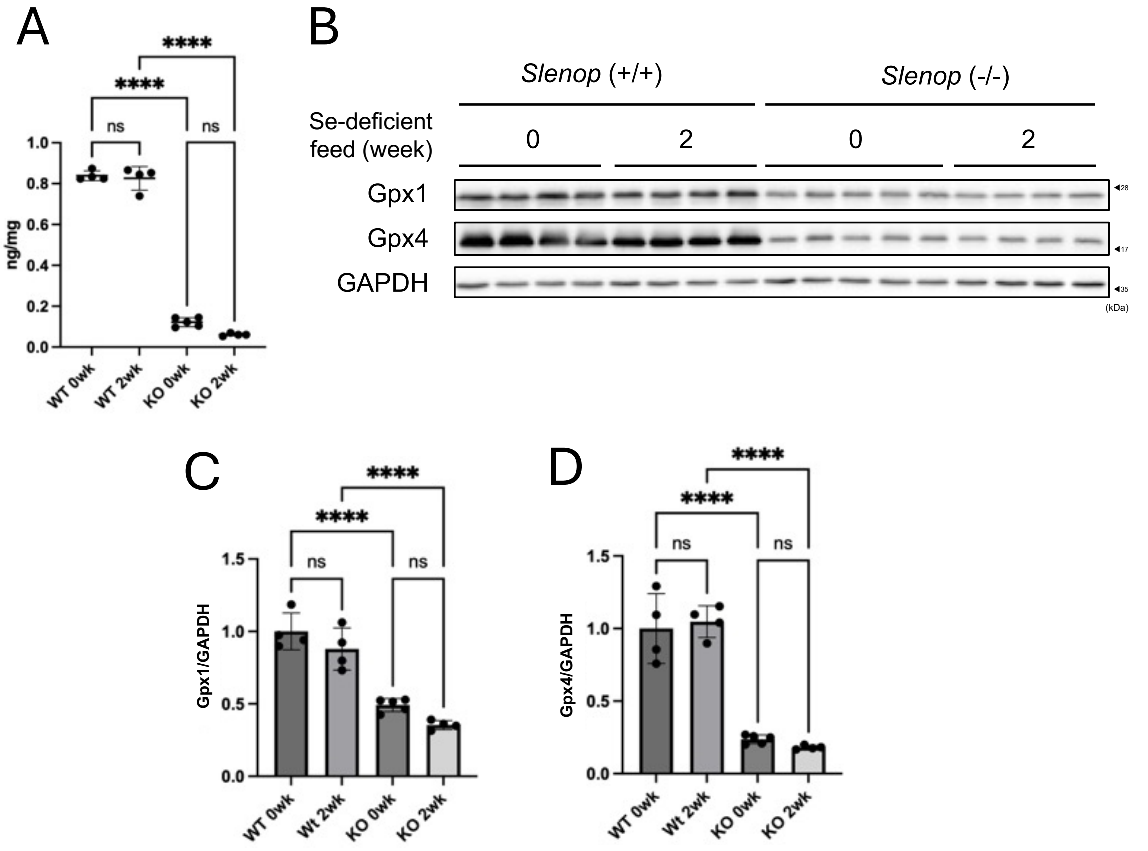


**Supplemental Figure 4. Effect of Se-deficiency on testis in mice.** WT and SeP KO mice grown on normal diet (CE2) were fed a Se-deficient diet for 2 weeks. **(A)** Total Se in testis were evaluated by ICP-MS**.** Mean ± S.D., n=4-5, *P<0.05, **P<0.01, Tukey'test. **(B)** WB of testis of WT or SeP KO mice shown in. **(C, D)** Quantitative values of the (C) Gpx1 and (D) Gpx4 bands were corrected for each GAPDH. Mean ± S.D., n=4-5, *P<0.05, **P<0.01, Tukey'test.

# Supplemental Figure 5

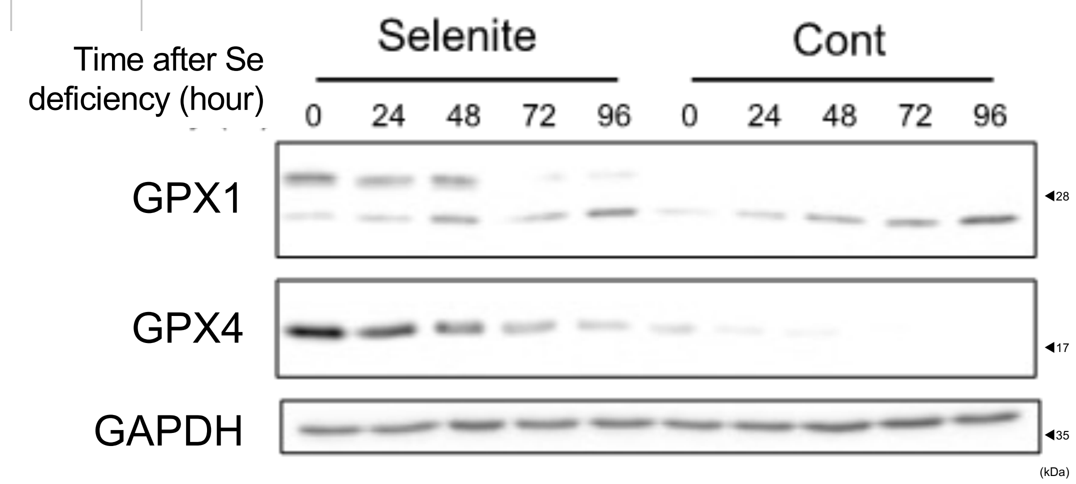


**Supplemental Figure 5. Effect of Se-deficiency on GPX expression in RD cells.** RD cells were treated with 100 nM of selenite for 24 hr and the medium was changed to a Se-deficient medium, then cells were cultured for the indicated period.

# Supplemental Figure 6

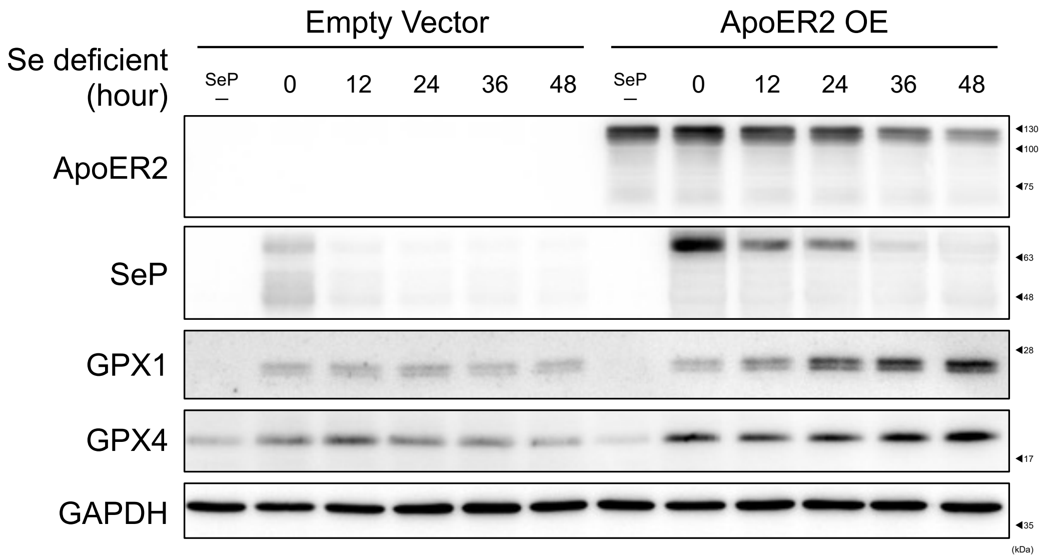


**Supplemental Figure 6. ApoER2 over-expression retains Se-metabolism by SeP under Se-deficient condition.** RD cells were transfected with empty vector or ApoER2 expressing plasmid (ApoER2 OE), then the cells were treated with SeP (0.5 µg/mL) for 24 hr. The cells were washed and the medium was changed to a Se-deficient medium and incubated for the indicated period.

# Supplemental Figure 7

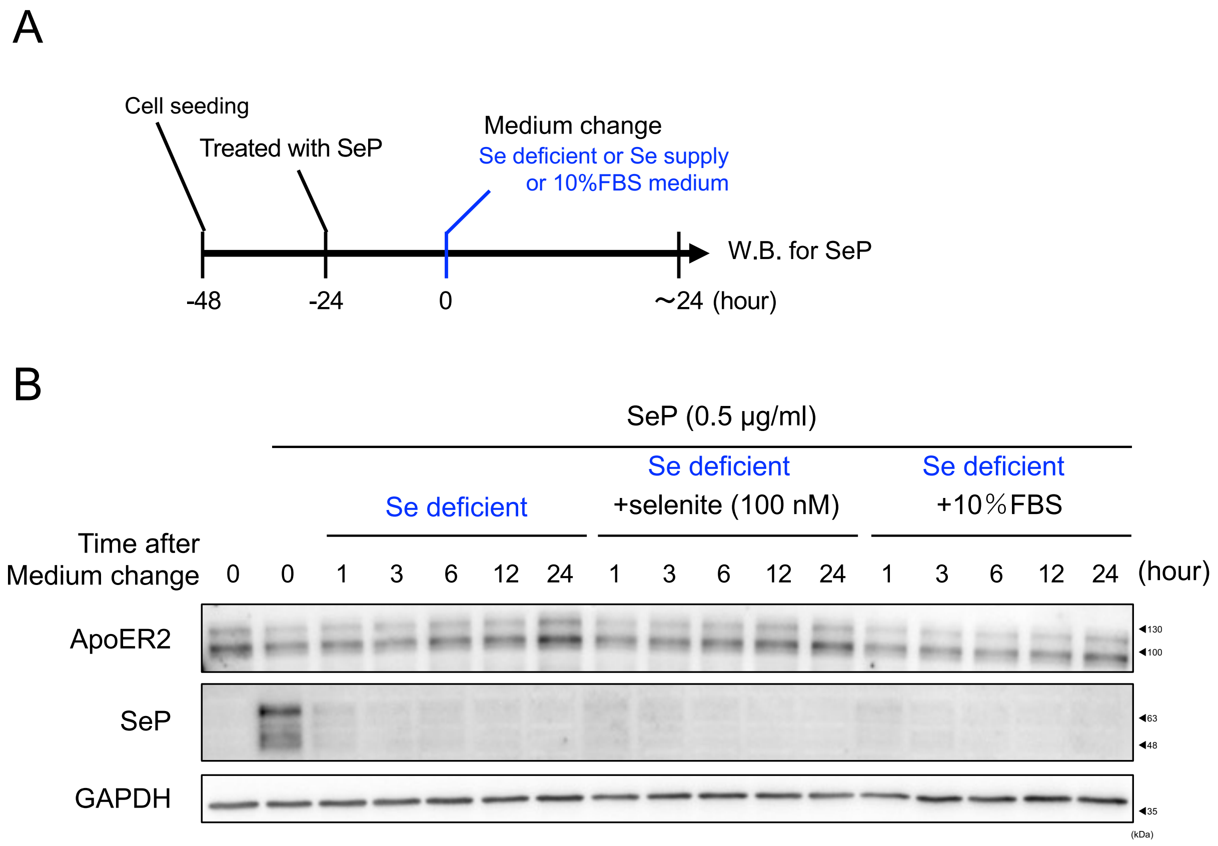


**Supplemental Figure 7. Effect of extracellular Se on the degradation of SeP in the cells. (A)** Experimental design. **(B)** RD cells were treated with SeP for 24 hr and then the medium was changed to Se-deficient medium or Se-deficient with selenite (100 nM) or Se-deficient with 10% FBS. Intracellular SeP levels were determined by WB.

# Supplemental Figure 8

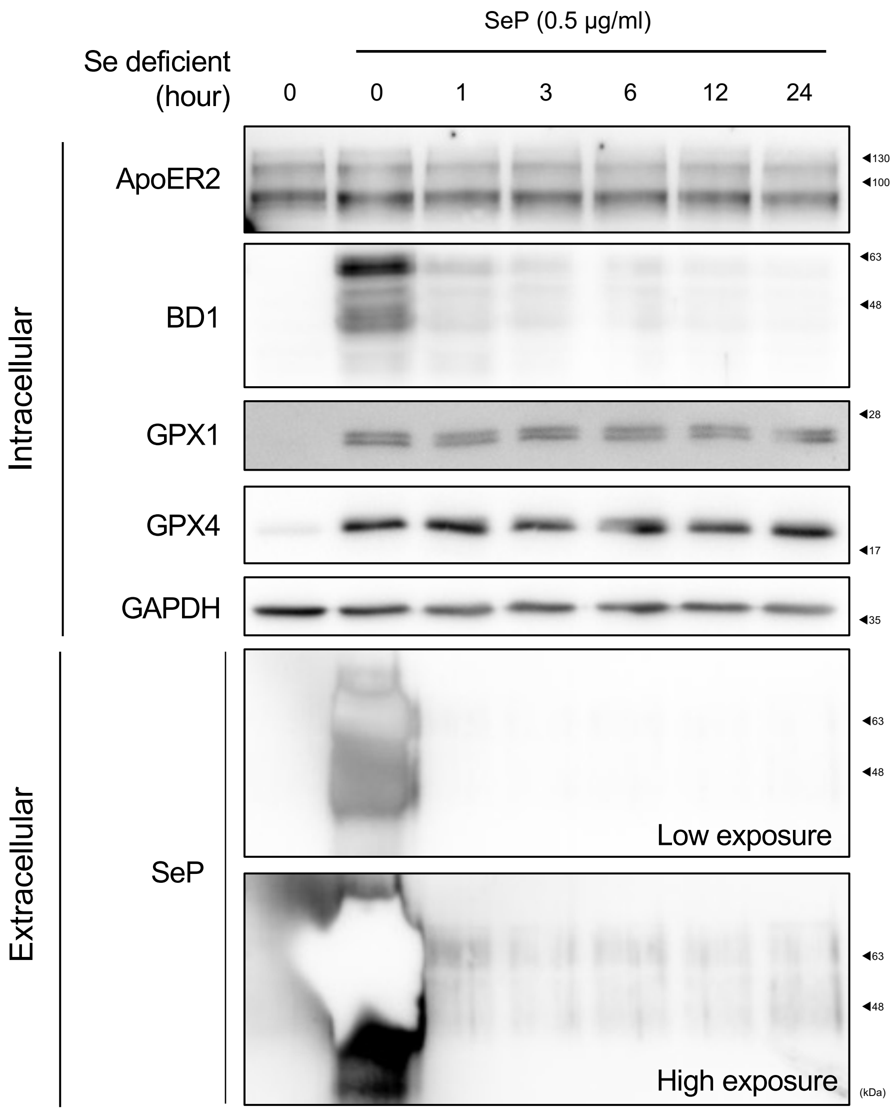


**Supplemental Figure 8. Re-release of SeP from SeP-charged cells. (A)** RD cells were treated with 0.5 µg/mL of SeP for 24 hr. After that, the medium was changed to a Se-deficient medium, and the medium was collected, and the SeP was concentrated by Ni-agarose. The elution was applied to WB as Extracellular.

# Supplemental Figure 9

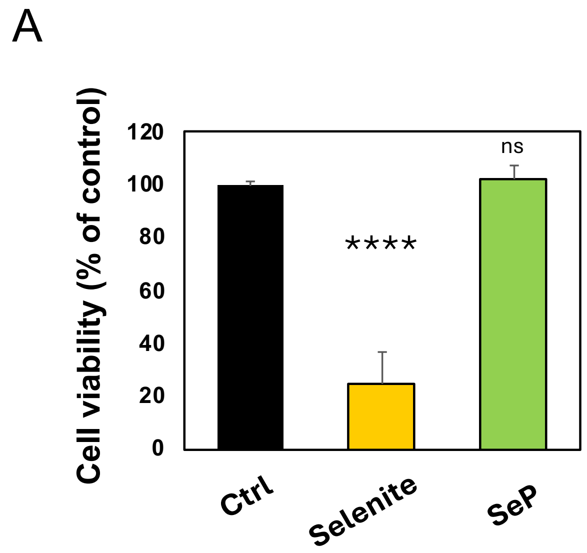


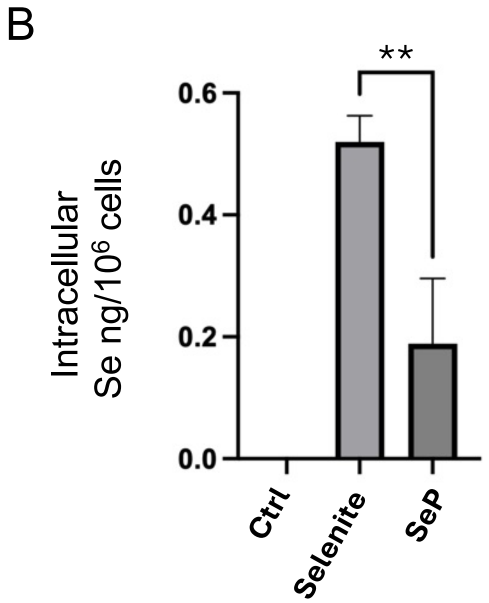


**Supplemental Figure 9. Re-release of SeP from SeP-charged cells.** RD cells were treated with 10 µM of selenite or 41.6 µg/mL of SeP. After 48 hr, (A) cell viability was measured by alamarBlue, and **(B)** selenium intake was evaluated by ICP-MS. Mean ± S.D., n=3, *P<0.05, **P<0.01, Tukey'test.

# Supplemental Figure 10

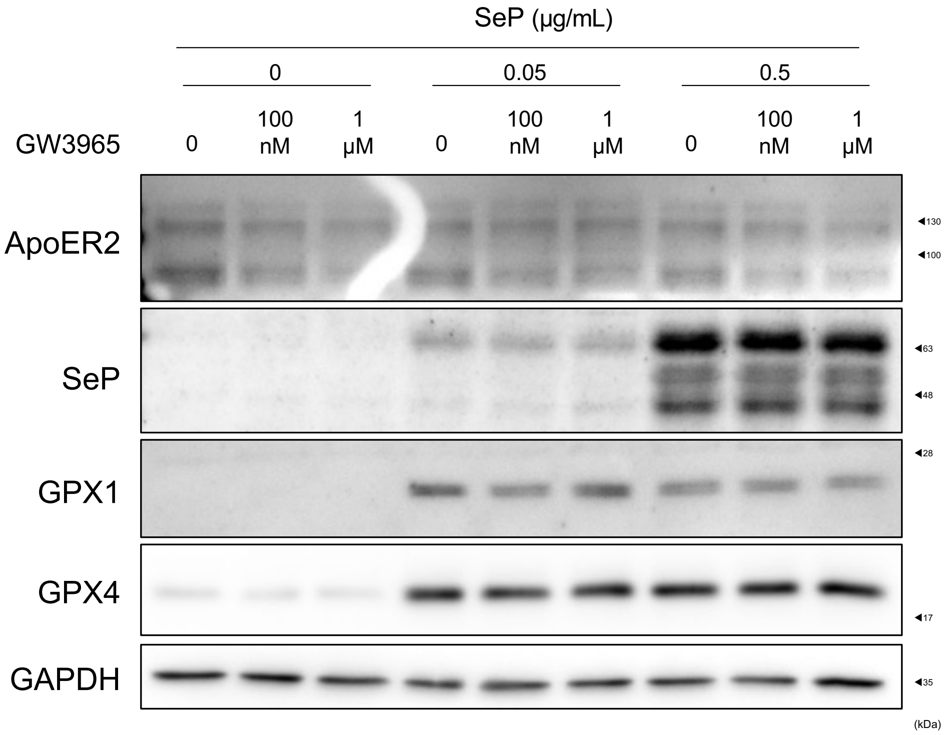


**Supplemental Figure 10. Effect of LXR agonist on ApoER2 expression and SeP incorporation.** RD cells were treated with GW3965, a LXR agonist, for 24 hr. Then the SeP were added and further icubated for 24 hr. ApoER2 expression and SeP incorporation were evaluated by WB.

# Supplemental Figure 11

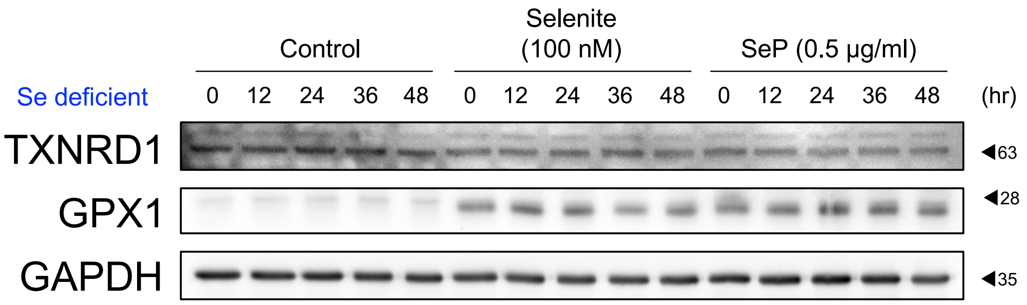


**Supplemental Figure 11. Effect of LXR agonist on ApoER2 expression and SeP incorporation.** RD cells were treated with selenite 100 nM and SeP 0.5 µg/mL for 24 hours. The medium was replaced with Se-depleted medium and further incubated for indicated time period. After that the cells were harvested and WB performed.
